# Understanding Resilience in Parents: Longitudinal Examination of Trait Resilience, Stressful Life Events, and Psychological Distress Symptoms—Insights From the FinnBrain Study

**DOI:** 10.1002/smi.3516

**Published:** 2024-12-02

**Authors:** Viivi Mondolin, Hasse Karlsson, Laura Perasto, Jetro J. Tuulari, Linnea Karlsson, Eeva‐Leena Kataja

**Affiliations:** ^1^ FinnBrain Birth Cohort Study Department of Clinical Medicine Turku Brain and Mind Center University of Turku Turku Finland; ^2^ Centre for Population Health Research Turku University Hospital University of Turku Turku Finland; ^3^ Department of Psychology and Speech‐Language Pathology University of Turku Turku Finland; ^4^ Department of Child Psychiatry Helsinki University Hospital University of Helsinki Helsinki Finland; ^5^ Pediatric Research Center New Children's Hospital Helsinki Finland; ^6^ Department of Psychiatry Turku University Hospital University of Turku Turku Finland; ^7^ Turku Collegium for Science, Medicine and Technology University of Turku Turku Finland; ^8^ Department of Public Health University of Turku and Turku University Hospital Turku Finland; ^9^ Department of Child Psychiatry Turku University Hospital University of Turku Turku Finland

**Keywords:** fathers, longitudinal study, mothers, stressful life events, trait resilience

## Abstract

The study aimed to investigate the persistence or changes in trait resilience of parents over a 6‐year period and its association with stressful life events (SLEs). Furthermore, we explored the potential protective effect of trait resilience against exposure to stressful life events and their negative mental health consequences. The study population was drawn from the ongoing FinnBrain Birth Cohort Study and included 1388 mothers and 657 fathers who completed the CD‐RISC‐10 questionnaire during pregnancy and again 6 years later. Data collection involved self‐report questionnaires, including CD‐RISC‐10, EPDS, SCL‐90, and a questionnaire on SLEs. Data analysis utilised linear regression and statistical assessments. Parents in the highest or lowest quartile of resilience showed greater stability in resilience scores over time compared to those in the middle quartiles. Trait resilience during pregnancy was significantly associated with resilience 6 years later. SLEs did not moderate this association. Additionally, higher trait resilience consistently associated with lower levels of distress symptoms. The investigation of SLEs may require more nuance due to their event‐specific variability of impact. Furthermore, the study's sample size of individuals who experienced a high frequency of stressful life events was limited. Trait resilience appears to be rather stable, but also susceptible to some change. Because of its persistency and the positive impact on mental health it is worthwhile to be assessed as a part of comprehensive evaluation of parents' mental health.

## 1 Introduction

Pregnancy and the early years of a child's life are a pivotal period of development for parents, and can be stressful and demanding (Deave, Johnson, and Ingram 2008). Child development progresses rapidly, and parents must continuously adapt to new situations which can be rather stressful as such. Parenting inherently encompasses a multitude of stressors, such as role overload, parenting guilt, and negative child behaviour, which are associated with elevated levels of depression, anxiety, and stress in mothers (Luthar and Ciciolla 2015). Furthermore, recent research indicates that Finnish parents exhibit higher levels of burnout compared to parents in many other countries worldwide (Roskam et al. 2021). In general, parental mental illnesses, including affective disorders, anxiety disorders and posttraumatic stress disorder, are relatively common, with estimates ranging between 10% and 38% (Ertel, Rich‐Edwards, and Koenen 2011; Reupert and Maybery 2016; van Santvoort et al. 2015). In the present study, the assessment of depressive and anxiety symptoms will be used as an indicator of psychological distress.

In addition to the stressors related to parenting, parents face various stressful life events and adversities in other domains of life, which, in some situations, can lead to significant distress and even mental disorders (Bjorndal et al. 2023; Ding et al. 2023; Kendler et al. 2010). Stressful life events (SLEs) are a variety of events and experiences, that are likely to cause some harm or threat to an individual, for example, death of a spouse, losing a job, having an injury or illness, or getting divorced (Cohen, Murphy, and Prather 2019). There is a broad consensus that stressful life events are associated with many types of problems, including psychological distress symptoms and psychiatric disorders (Cohen, Murphy, and Prather 2019; Hjemdal et al. 2006; Kendler et al. 2010; Sheerin et al. 2018). The significance of stressful life events is underscored by research findings, which indicate that individuals who experience depression are 2.5–9.4 times more prone to having encountered a major stressful life event preceding their first depression episode (Cohen, Murphy, and Prather 2019). The pathways through which stressful life events affect mental health are diverse. It is widely accepted that such events can increase the risk of illness by affecting an individual's emotions, behaviour, and physiology (Cohen, Murphy, and Prather 2019). However, although there is a well‐established connection between SLEs and mental health problems, most people do not become ill when facing adversities. While the exact mechanisms by which SLEs lead to illness are not fully understood, there appear to be some key factors that may protect against the negative outcomes of SLEs. One such factor may be resilience.

The field of resilience research has been impeded by inconsistent and sometimes vague definitions and conceptualisations of resilience (Davydov et al. 2010; Fletcher and Sarkar 2013). This lack of coherence has hindered research findings and the development of practical applications, particularly in mental health (Hu, Zhang, and Wang 2015). Although recent advancements have led to more sophisticated and precise definitions of resilience, there remains considerable variation in its interpretation. One potential explanation for the diverse definitions and operationalisation of resilience is the inherent complexity of the phenomenon itself. Ungar and Theron (2020) have demonstrated the value of a multisystemic approach in the field of resilience science. They elucidate that resilience is optimally conceived as a process entailing the interplay of multiple biological, psychological, social, and ecological systems that assist individuals in recovering, maintaining, or enhancing their mental well‐being when confronted with one or more risk factors (Ungar and Theron 2020). A frequently utilised conceptualisation of resilience is that it is a dynamic process involving positive adaptation within the context of significant adversity (Fletcher and Sarkar 2013; Luthar, Cichetti & Becker 2000). The aforementioned theory of Ungar and Theron (2020) provides an illustrative example of the manner in which a multiplicity of factors can be related to and affect the resilience. Furthermore, it is a common research approach to define resilience as an outcome. This is where resilience is defined as a function or behavioural outcome that enables individuals to effectively overcome and recover from adversities (Hu, Zhang, and Wang 2015; Nishimi et al. 2020). These perspectives stipulate that two conditions must be met for resilience to exist: there must be an observable risk factor and a positive outcome (Luthar, Cicchetti, and Becker 2000; Masten 2001).

Another prevalent conceptualisation of resilience is as a personal trait that enables individuals to effectively cope with challenges and achieve positive adaptation (Connor and Davidson 2003; Hu, Zhang, and Wang 2015). Those who espouse this perspective regard resilience as a protective factor that enables individuals to endure and navigate adversities or traumatic experiences (Connor and Davidson 2003; Ong et al. 2006). This trait encompasses qualities such as toughness and perseverance, which are primarily evaluated through self‐report questionnaires (Campbell‐Sills and Stein 2007; Connor and Davidson 2003). These assessments quantify an individual's capacity to adapt to changes, cope with unexpected events or illnesses, and recover from setbacks, thereby enabling them to overcome these challenges (Campbell‐Sills and Stein 2007). The trait resilience has been utilised in clinical settings (Connor and Davidson 2003; Dai et al. 2024) to provide a quantifiable and measurable score that can be tracked and operationalised. Furthermore, the concept of resilience as a trait has been the subject of debate and criticism. A significant point of contention has been the implication that resilience is a fixed trait (Luthar, Cicchetti, and Becker 2000; Windle 2011). Additionally, some have suggested that the absence of trait resilience could be perceived as a deficit or failure of the individual (Windle 2011).

Trait resilience has been frequently associated with lower levels of stress, depression, and anxiety in different age groups (Anyan and Hjemdal 2016; Cheng et al. 2020; García‐León et al. 2019; Hu, Zhang, and Wang 2015; Laird et al. 2019). In both the pregnant and postpartum periods, low levels of resilience have been associated with an increased prevalence of depressive symptoms, depressive and anxiety disorders, and post‐traumatic stress disorder (Kinser et al. 2021; Mondolin et al. 2024; Osofsky et al. 2021; Sexton et al. 2015; Young‐Wolff et al. 2019). Resilience has also been found to provide protection from the harmful effects of adverse early life experiences among parents (Osofsky et al. 2021; Sexton et al. 2015; Young‐Wolff et al. 2019). Additionally, self‐reported resilience among Finnish parents has been identified as a protective factor against parental burnout (Sorkkila and Aunola 2022). Few studies have explored the relationship between resilience and SLEs, with results indicating the protective effects of resilience against the negative impacts of these events. In a study by Hjemdal et al. (2006) self‐reported resilience served as a buffer against the psychiatric symptoms in the face of SLEs. In a study with a 5‐year follow‐up period, individuals with high levels of resilience were less likely to develop major depressive disorder (MDD) or generalised anxiety disorder (GAD), even when faced with a high frequency of SLEs in the past year (Sheerin et al. 2018). Furthermore, individuals with higher trait resilience have been found to not only experience a reduced impact of perceived stress related to SLEs, but also report fewer stressful experiences overall (García‐León et al. 2019).

Despite the growing body of research on trait resilience and its acknowledged significance for mental health, questions remain about its fundamental characteristics, such as its stability over time. While often perceived as an enduring characteristic, implying its lasting nature, trait resilience may also exhibit susceptibility to change. Trait resilience has been part of several longitudinal studies, although its stability has not been the primary research question. For example, a 4‐year follow‐up study on resident physicians revealed a decrease in trait resilience among women, but not men (Perry et al. 2021). In another study of medical students, relative stability was found over a 20‐month period (Q. Wang et al. 2022). A 2‐year follow‐up study conducted during and after the COVID‐19 pandemic observed a slight decrease in resilience (Sumner et al. 2023). Furthermore, there is evidence, supported by at least one cross‐sectional study, that trait resilience may increase with age (Lundman et al. 2007). As noted in a systematic review by Cosco et al. (2017), there is a dearth of comprehensive longitudinal studies that focus on resilience as a primary outcome. The existing studies have not thoroughly explored how resilience persists or changes over time and interacts with events and behaviours (Cosco et al. 2017). The present study aims to address, to some extent, the aforementioned gap in the current literature.

In this study, the conceptualisation of trait resilience is employed due to its significant relevance to mental health issues, as previously discussed. The availability of a well‐established measurement tool and its potential clinical applications are also of great importance from our perspective. It is important to note that resilience is not assumed to be a fixed entity, as evidence suggests it can be modified (Dai et al. 2024; J. Davidson et al. 2008). However, it is expected that resilience will remain stable to a certain extent. Moreover, it is postulated that trait resilience may serve to mitigate the negative impact of stressful life events. Additionally, it is recognised that trait resilience is only one aspect of the broader concept of resilience, as illustrated by the multi‐system approach proposed by Ungar and Theron (2020). To this end, the present study will focus on two specific areas: first, the long‐term stability of trait resilience; and second, the relationship between trait resilience, stressful life events and psychological distress (depressive and anxiety symptoms). The perinatal period and early parenthood provide an optimal setting for investigating trait resilience, as this time is characterised by relentless change and numerous challenges. Furthermore, research conducted during this period is of paramount importance due to its profound impact on the health and well‐being of both the next generation and the entire family. Consequently, studying trait resilience and mental health during this time is vital, with research into the potential value of trait resilience in this context just beginning to emerge.

The research questions are summarised in three hypotheses. Hypothesis 1 states that there is a tendency for trait resilience to persist over the 6‐year longitudinal period, with parents demonstrating a consistent trend in their scores. Hypothesis 2 suggests that stressful life events may serve to moderate the associations between trait resilience during pregnancy and trait resilience 6 years later, potentially resulting in a weakening of the relationship. Hypothesis 3 predicts that trait resilience will act as a protective factor against distress symptoms associated with stressful life events. Consequently, parents who exhibit high levels of trait resilience are anticipated to demonstrate superior coping abilities in the face of challenging life circumstances, resulting in a reduction in the prevalence of distress symptoms.

## 2 Methods

This research is a part of the ongoing FinnBrain Birth Cohort Study (www.finnbrain.fi), which aims to investigate the effects of early life stress, including prenatal distress, on the development of a child's brain and health in a prospective manner. The study protocol has been approved by the Ethics Committee of the Hospital District of Southwest Finland.

### 2.1 Participants

Participants were recruited during their first ultrasound appointment at 12 weeks' gestation (gwk) at three maternal welfare clinics in southwestern Finland between December 2011 and April 2015. The study's inclusion criteria required a sufficient command of either Finnish or Swedish (the official languages of Finland), as well as a normal ultrasound screening result. After receiving information on the study's aims and procedures, parents provided written consent. Questionnaires were used to collect data from 3808 mothers and 2623 fathers during pregnancy. The study cohort consisted mainly of ethnically homogeneous Scandinavian Caucasian individuals and was broadly representative of the expectant parent population in Southwest Finland (Karlsson et al. 2018). The inclusion criteria for this study were that participants in the study cohort had completed the Connor‐Davidson Resilience Scale 10 (CD‐RISC‐10) questionnaire twice, initially at the first time point (T1) at gwk 14 and subsequently at the second time point (T2) 6 years later. At the initial measurement, 3034 mothers completed the CD‐RISC‐10 survey. Six years later, 1390 mothers participated in the follow‐up survey, indicating attrition in respondents. Initially, 1950 fathers responded to the CD‐RISC‐10 questionnaire, and after 6 years, only 685 participated. Finally, the present study group consisted of 1388 mothers and 657 fathers who had completed both CD‐RISC‐10 questionnaires at time T1 and at T2.

An attrition analysis was conducted to compare the group of participants who responded at both T1 and T2 with the group of participants who responded only at T1. Among mothers, those who did not respond were younger (*M* = 30.0 vs. 30.9, *p* < 0.001) and had lower educational attainment (*p* < 0.001) and lower income levels (*p* < 0.001) compared to the responding mothers. Furthermore, mothers who did not respond exhibited lower trait resilience (*M* = 27.7 vs. 28.3, *p* < 0.001) and higher levels of depressive symptoms (*M* = 5.5 vs. 4.8, *p* < 0.001) and anxiety symptoms (*M* = 3.5 vs. 3.1, *p* < 0.05). Among fathers, non‐respondents were younger (*M* = 31.9 vs. 32.6, *p* < 0.05), had lower education levels (*p* < 0.001), and reported higher levels of depressive symptoms (*M* = 3.9 vs. 3.5, *p* < 0.05). Despite these differences, due the high attrition rates observed, which exceeded over 50% for both mothers and fathers, we decided not to impute the missing data. Though the attrition analysis yielded statistically significant discrepancies, these differences were relatively minor when the descriptive statistics were examined.

Table 1 displays the characteristics of the sample. The participants' educational attainment was classified into three categories in accordance with the Finnish school system. The categories were defined as follows: less than 12 years represents basic education, 12–15 years corresponds to secondary education, and any level of education beyond that is classified as higher education. Monthly net income was divided into four categories for the purpose of facilitating interpretation: < 1500€, 1501€–2500€, 2501€–3500€, and over 3500€. The stressful life events (SLEs) were classified into three categories: those who had not reported any SLEs, those with a single event, and those who had experienced two or more SLEs.

**TABLE 1:** Characteristics of the study sample.

(*N* = Mothers, *N* = Fathers)		Mothers	Fathers
		*M*, (SD; range)	*M*, (SD; range)
Age at enrolment, mean (1388, 657)		30.9 (4.2; 18–45)	32.5 (5.2; 21–57)
Education, (1386, 656)		2.1 (0.8; 1–3)	2.0 (0.8; 1–3)
Education, (%)	< 12 years	28.7	34.8
	12–15 years	30.2	32.2
	> 15 years	41.1	33.1
Net income, €/month, (1383, 653)		1.8 (0.7; 1–4)	2.1 (0.8; 1–4)
Net income, €/month (%)	≤ 1500	34.7	19.3
	1501–2500	54.4	54.2
	2501–3500	9.3	22.1
	> 3500	1.7	4.4
Questionnaire scores, mean (SD; range)			
Resilience T1 (1388, 657)		28.3 (4.9; 5–40)	29.3 (5.2; 10–40)
Resilience T2 (1388, 657)		29.1 (6.0; 0–40)	29.8 (5.2; 4–40)
Depressive symptoms T1 (1385, 656)		4.8 (3.9; 0–27)	3.5 (3.3; 0–22)
Depressive symptoms T2 (1381, 654)		5.0 (4.5; 0–26)	4.1 (4.1; 0–23)
Anxiety symptoms T1 (1386, 655)		3.1 (3.8; 0–33)	2.4 (3.2; 0–22)
Anxiety symptoms T2 (1378, 653)		4.2 (5.0; 0–37)	3.0 (3.9; 0–29)
Stressful life event continuous (1388, 657)		1.1 (1.4; 0–9)	0.7 (1.1; 0–9)

Stressful life event categorised, mean (SD)	Mothers			Fathers		
	0	1	≥ 2	0	1	≥ 2
*N* (%)	656 (47.3)	357 (25.7)	375 (27.0)	398 (60.6)	149 (22.7)	110 (16.7)
Resilience T2	29.7 (5.8)	28.6 (6.5)	28.7 (6.0)	30.2 (5.6)	29.6 (5.4)	28.4 (6.1)
Depressive symptoms T2	4.2 (4.1)	5.6 (4.8)	5.9 (4.7)	3.8 (3.8)	3.6 (3.7)	5.9 (5.1)
Anxiety symptoms T2	3.4 (4.4)	4.8 (5.4)	4.9 (5.3)	2.7 (3.6)	2.8 (3.7)	4.4 (5.0)

*Note:* Age, education and income are gathered in the measurement point 1 (T1). Resilience = CD‐RISC‐10, Depressive symptoms = EPDS, Anxiety symptoms = SCL 90 (anxiety subscale), Stressful life events = SLE sum score from the whole follow‐up period.

### 2.2 Measures

The study measures were collected over a follow‐up period of 6 years. The first measurement point (T1) included measures of trait resilience and depressive and anxiety symptoms. Data on stressful life events (SLEs) were collected at five different time points: 6 months postpartum, 1, 2 and 4 years after delivery, and at the final time point (T2) 5 years after delivery. In addition to the SLEs, data on trait resilience, depressive symptoms and anxiety symptoms were also collected at T2. A more comprehensive account of the measurements can be found in the subsequent sections.

#### 2.2.1 Trait Resilience

Resilience was assessed using the Connor Davidson Resilience Scale 10 (CD‐RISC‐10), a self‐report instrument with robust psychometric properties (Cheng et al. 2020; Connor and Davidson 2003; L. Wang et al. 2010). The CD‐RISC‐10 assesses an individual's perception of their ability to navigate and cope with adversity. It is comprised of 10 items, such as the ability to adapt to change, responses to difficulties, recovery after illness or injury, and persistence in the face of failure. The scale employs a 5‐point Likert scale (ranging from 0, ‘not at all true’, to 4, ‘true almost all of the time’) to generate a composite score between 0 and 40, whereby higher scores indicate greater resilience. The internal consistency of the CD‐RISC‐10 was strong for mothers with *α* = 0.84 (T1) and *α* = 0.90 (T2) and for fathers with *α* = 0.85 (T1) and *α* = 0.88 (T2).

#### 2.2.2 Stressful Life Events (SLEs)

**FIGURE 1:**
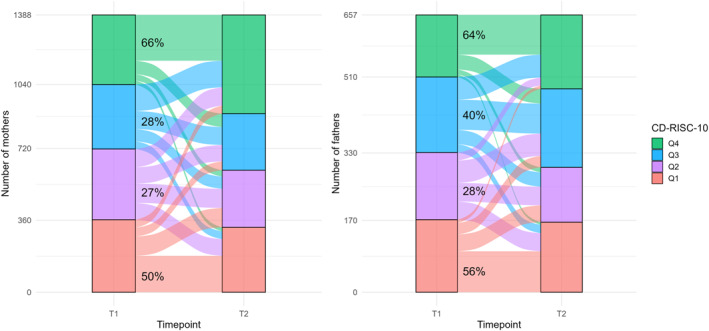
The stability percentages for trait resilience (CD‐RISC‐10) are presented by quarter, with Q1 indicating the lowest and Q4 indicating the highest. The percentages represent the number of individuals who remained in the same quarter at the 6‐year follow‐up point.

We selected 9 items from a questionnaire of 17 life events to assess stressful life events (SLEs). The SLEs were assessed at five different time points (see Figure 1). The questions were derived from previous studies conducted in Finland (Vahtera et al. 2007), with adaptations to better fit the specific focus of our study on parents during pregnancy and the early years of their children's lives. We modified certain elements of the questionnaire and included additional questions related to parenthood, including supplementary questions on childcare and related factors. In summary, the assessment of SLEs comprises nine items: death of the child's parents, divorce/separation of the child's parents, serious illness of the child, death of the child's grandparent, residential separation of the child's parents, parental unemployment, serious illness of the child's grandparent and deterioration in the family's financial situation. Data on life events were collected at five different time points, with participants responding whether they had experienced any of the events within the past year (or within the last 6 months for the 6‐month questionnaire). Responses were recorded as either ‘yes’ or ‘no’ for each item at each time point, and a total frequency score was calculated for each participant by summing these responses across the entire follow‐up period.

#### 2.2.3 Psychological Distress

We assessed the level of psychological distress experienced by mothers and fathers by administering questionnaires assessing symptoms of depression and anxiety during pregnancy at 14 weeks, with follow‐up assessments 6 years later. Depressive symptoms were evaluated using the Edinburgh Postnatal Depression Scale (EPDS) (Cox, Holden, and Sagovsky 1987), a validated tool designed to detect antenatal depression objectively across different cultural and geographical contexts (Matijasevich et al. 2015; Rubertsson et al. 2011). The questionnaire comprises 10 questions scored on a 4‐point Likert scale (0, 1, 2, or 3), with total scores ranging from 0 to 30. The EPDS demonstrated sufficient internal consistency with *α* = 0.82 (T1) and *α* = 0.86 (T2) for mothers with *α* = 0.78 (T1) and *α* = 0.85 (T2) for fathers. Anxiety symptoms were assessed using the Symptom Checklist (SCL‐90), specifically the anxiety subscale consisting of 10 items (Derogatis, Lipman, and Covi 1973). Participants rated these items on a 5‐point Likert scale ranging from 0 to 4 points, resulting in total scores ranging from 0 to 40 points. The reliability of the subscale has been confirmed in the Finnish population (Holi, Sammallahti, and Aalberg 1998). In this study, the results suggest that the anxiety subscale has satisfactory internal consistency for mothers with *α* = 0.83 (T1) and *α* = 0.86 (T2) and for fathers with *α* = 0.80 (T1) and *α* = 0.84 (T2).

### 2.3 Statistical Analyses

Correlations between continuous resilience scores were first examined using the paired Wilcoxon signed‐rank test. To aid the analysis of hypothesis 1 (H1), resilience scores were divided into quarters, following a similar approach to previous studies (J. R. T. Davidson 2021; Mondolin et al. 2024). The quarterly division was based on the distribution of resilience scores at the initial measurement point (T1). The distribution of quarterly scores for mothers was as follows: Q1 for scores ≤ 25, Q2 for scores between 26 and 28, Q3 for scores between 29 and 31, and Q4 for scores > 31. The quarterly scores for fathers were distributed as follows: Q1 for scores ≤ 26, Q2 for scores between 27 and 29, Q3 for scores between 30 and 33, and Q4 for scores > 33. Cross‐tabulation and the Chi‐Square test were used to examine the stability of the categorised resilience scores at two time points.

In order to conduct an analysis of hypotheses 2 and 3, the SLE was divided into three categories, as the distribution was found to be too skewed when treated as a continuous variable. First the main effects of resilience in T1 and SLEs were examined using the following linear model:

Model1:resilienceT2∼intercept+resilienceT1+SLEs+education+age+EPDS,

where both resilience scores, age and EPDS T2 were continuous variables while SLEs (0, 1 event, 2 or more events) and education (low, mid, high) were categorical. Next moderation analysis was formulated by adding the interaction term between resilience T1 and SLEs to Model 1. As a sensitivity analysis, SCL‐90 in T2 was also included in the models. The assumptions of linear models were assessed visually and using the Shapiro–Wilk test.

In hypothesis 3, first the main effects of resilience T1 and SLEs were examined using the following linear model:

Model2:EPDS/SCL∼intercept+resilienceT1+SLEs+education+age,

where dependent variable was either continuous EPDS T2 or SCL T2 and other variables as in model 1. Next moderation analysis was formulated by adding the interaction term between resilience T1 and SLEs to Model 2. As a sensitivity analysis, EPDS T1 and SCL‐90 T1 was also included in the models. The distribution of model's residuals was skewed so the bias‐corrected and accelerated (BCa) (Efron 1987) bootstrap confidence intervals and bias‐corrected estimates were also calculated (based on 5000 bootstrap samples).

All analysis were done separately for mothers and fathers. *p*‐values (two‐tailed) smaller than 0.05 were interpreted as statistically significant. The beta coefficients (*b*) and 95% confidence intervals (CI) and partial Eta Squared (*η* ^2^) were calculated in hypotheses 2 and 3. In hypothesis 3 also the bootstrap confidence intervals (BCa CI) and bias‐corrected estimates (*b*_BC_) were calculated. The analyses were performed using R (4.2.2, 2022). Bootstrap was calculated using the library boot and figures were made using the libraries ggplot2, ggeffects and ggalluvial (Davison and Hinkley 1997; Lüdecke 2018; Wickham 2016). Finally, to provide an overview of the data, gender differences between mothers and fathers were examined using independent samples *t*‐tests. Welch's correction was applied when the assumption of homogeneity of variances was not met.

## 3 Results

### 3.1 Stressful Life Events in the Sample

The prevalence of SLEs is presented in Table 1, both as a continuous variable and as a categorical variable with three categories: 0, 1, or 2 or more SLEs. A total of 656 mothers (47.3%) and 398 fathers (60.6%) indicated that they had not experienced any SLEs. Furthermore, 357 mothers (25.7%) and 149 fathers (22.7%) reported one SLE, while 375 mothers (27.0%) and 110 fathers (16.7%) reported two or more SLEs. The most common SLE reported by parents was a decline in their family's financial situation. This was reported by 311 mothers and 122 fathers once, 164 mothers and 16 fathers twice, and 45 mothers and 7 fathers three or four times. Other frequently reported events included unemployment, serious illness of the child's grandparent, and the death of a child's grandparent.

### 3.2 The Stability of Trait Resilience

We analysed how trait resilience is maintained over a 6‐year follow‐up period. The trait resilience score increased slightly but significantly for mothers (from an average of 28.3–29.3) and fathers (from an average of 29.1–29.8). Correlational analyses revealed moderate to strong positive associations between trait resilience in T1 and T2 for mothers (rs = 0.54, *p* < 0.001) and fathers (rs = 0.63, *p* < 0.001). When examined by quartile, individuals exhibited more variability than stability in resilience: 43% of all mothers (*N* = 597) and 47% of all fathers (*N* = 306) remained in the same quartile. However, quartile‐specific analyses revealed disparities between categories: in the highest quartile (Q4), 66% of mothers and 64% of fathers remained, while in the lowest quartile (Q1), 50% of mothers and 56% of fathers remained. In contrast, in the middle quartiles, the remaining percentages were 27% in Q2 and 28% in Q3 for mothers, and 28% in Q2 and 40% in Q3 for fathers. Overall, the highest and lowest resilience groups demonstrated greater stability compared to the middle categories. The stability percentages for each quartile are presented in Figure 1.

Linear regression analysis revealed a significant direct relationship between T1 and T2 resilience scores for both mothers (*b* = 0.55, 95% CI [0.50; 0.60], *p* < 0.001) and fathers (*b* = 0.62, 95% CI [0.55; 0.68], *p* < 0.001), adjusting for depressive and anxiety symptoms, education and age. Furthermore, the potential moderating effect of stressful life events on this association was examined. However, we did not observe any interaction effect of SLEs on the relationship between resilience at T1 and T2 for either mothers (1 event: *b* = 0.01, 95% CI [−0.11; 0.13], *p* = 0.897; ≥ 2 events: *b* = −0.04, 95% CI [−0.15; 0.08], *p* = 0.508) or fathers (1 event: *b* = −0.01, 95% CI [−0.16; 0.14], *p* = 0.893; ≥ 2 events: *b* = −0.01, 95% CI [−0.15; 0.08], *p* = 0.508). The results of the regression analyses are presented in Table 2.

**TABLE 2:** The regression models of the trait resilience during pregnancy and trait resilience 6 years after, with interaction analysis of stressful life events.

	Mothers				Fathers			
	*b*	95% CI	*p*‐value	*η* ^2^	*b*	95% CI	*p*‐value	*η* ^2^
Main effects								
(Intercept)	15.02	12.80–17.24	< 0.001		14.83	12.09–17.56	< 0.001	
Resilience T1	0.55	0.50–0.60	< 0.001	0.255	0.62	0.55–0.68	< 0.001	0.372
SLEs (1 event)^a^	−0.09	−0.67–0.50	0.768	0.001	−0.18	−0.93–0.58	0.648	0.001
SLEs (≥ 2 events)^a^	0.46	−0.11–1.04	0.115		0.12	−0.74–0.98	0.785	
Education (mid)^b^	0.01	−0.62–0.64	0.974	0.001	0.11	−0.64–0.87	0.766	< 0.001
Education (high)^b^	−0.34	−0.94–0.27	0.275		−0.01	−0.77–0.74	0.973	
Age	0.04	−0.02–0.10	0.189	0.001	−0.04	−0.10–0.02	0.225	0.002
EPDS T2	−0.53	−0.58 to −0.47	< 0.001	0.207	−0.46	−0.54 to −0.38	< 0.001	0.164
Moderation								
Resilience T1 × SLEs (1 event)^a^	0.01	−0.11–0.13	0.897	< 0.001	−0.01	−0.16–0.14	0.893	< 0.001
Resilience T1 × SLEs (≥ 2 events)^a^	−0.04	−0.15–0.08	0.508		−0.01	−0.16–0.14	0.877	

*Note:* The table only displays estimates for the interaction term in the moderation analysis. The moderation analysis included the same variables as the main effects model.

^a^ The reference level is 0 events.

^b^ The reference level is low.

### 3.3 Resilience as a Protective Factor

**FIGURE 2:**
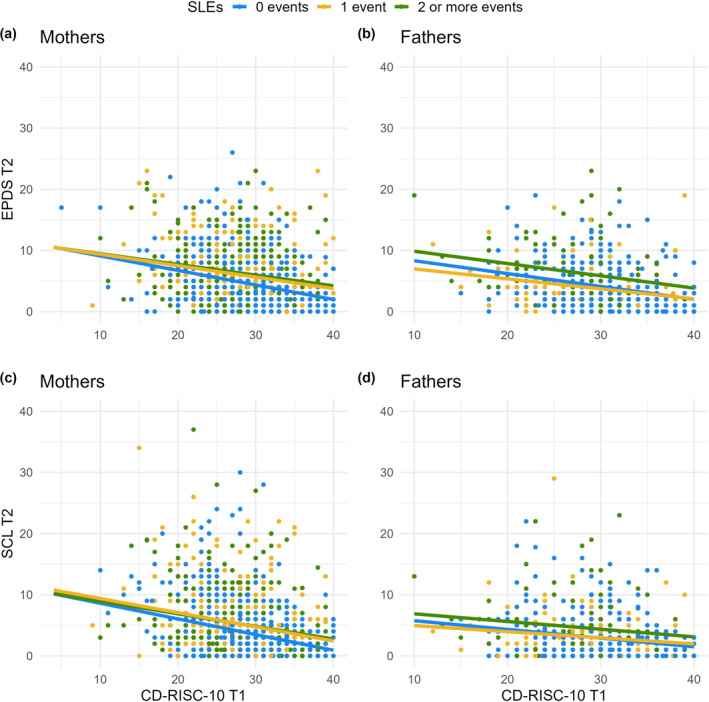
Associations between trait resilience (CD‐RISC‐10) at time point 1 (T1), stressful life events (SLEs) score accumulated over the entire follow‐up period, depressive symptoms (EPDS), and anxiety symptoms (SCL, anxiety subscale) at the end of the follow‐up period (time point 2, T2) in mothers and fathers, based on the interaction models. Panel (a) depicts the association between trait resilience and depressive symptoms in mothers who have experienced zero, one, or more stressful life events. Panel (b) illustrates the analogous association for fathers. Panel (c) depicts the association between trait resilience and anxiety symptoms in mothers with zero, one, or more stressful life events, while panel (d) illustrates the same for fathers.

Finally, it was investigated whether resilience has the potential to protect against the adverse effects of stressful life events, as indicated by an increase in depressive and anxiety symptoms. A statistically significant main effect was found between SLEs and symptoms of depression for mothers (1 event*: b*_BC_ = 1.19, 95% BCa CI [0.65; 1.79], *p* < 0.001; ≥ 2 events: *b*_BC_ = 1.49, 95% BCa CI [0.94; 2.06], *p* < 0.001) and fathers (≥ 2 events: *b*_BC_ = 1.74, 95% BCa CI [0.82; 2.78], *p* < 0.001) at T2. Furthermore, SLEs and anxiety were found to be associated in mothers (1 event: *b*_BC_ = 1.23, 95% BCa CI [0.63; 1.89], *p* < 0.001; ≥ 2 events: *b*_BC_ = 1.25, 95% BCa CI [0.64; 1.88], *p* < 0.001) and fathers (≥ 2 events: *b*_BC_ = 1.45, 95% BCa CI [0.55; 2.51], *p* < 0.001) at T2. In mothers, the presence of one or more SLEs was associated with an increased prevalence of psychological distress, whereas among fathers, only the presence of two or more SLEs increased symptoms of distress. Higher resilience assessed during pregnancy (T1) was associated with lower levels of depressive symptoms (mothers *b*_BC_ = −0.21, 95% BCa CI [−0.26; −0.15], *p* < 0.001; fathers *b*_BC_ = −0.20, 95% BCa CI [−0.25; −0.13], *p* < 0.001) and anxiety symptoms (mothers *b*_BC_ = −0.24, 95% BCa CI [−0.30; −0.18], *p* < 0.001; fathers *b*_BC_ = −0.13, 95% BCa CI [−0.19; −0.08], *p* < 0.001) at T2. This association remained significant for mothers, even after controlling for depressive and anxiety symptoms at T1, but not for fathers. However, contrary to our hypothesis, resilience did not moderate the relationship between SLEs and depressive symptoms in mothers (1 event: *b*_BC_ = 0.05, 95% BCa CI [−0.10; 0.19], *p* = 0.389; ≥ 2 events: *b*_BC_ = 0.06, 95% BCa CI [−0.06; 0.19], *p* = 0.268) or fathers (1 event: *b*_BC_ = 0.05, 95% BCa CI [−0.08; 0.22], *p* = 0.517; ≥ 2 events: *b*_BC_ = 0.01, 95% BCa CI [−0.16; 0.16], *p* = 0.904). The finding were similar when considering SLEs and anxiety in mothers (1 event: *b*_BC_ = 0.02, 95% BCa CI [−0.15; 0.17], *p* = 0.698; ≥ 2 events: *b*_BC_ = 0.05, 95% BCa CI [−0.09; 0.17], *p* = 0.423) and fathers (1 event: *b*_BC_ = 0.04, 95% BCa CI [−0.09; 0.18], *p* = 0.553; ≥ 2 events: *b*_BC_ = 0.02, 95% BCa CI [−0.14; 0.16], *p* = 0.826). In other words, the effect of trait resilience on distress symptoms remained consistent and appeared to provide equal protection, regardless of whether a person had experienced 0, 1 or 2 or more SLEs (see Figure 2). The results of the regression analyses are shown in Table 3.

**TABLE 3:** The regression models of the SLEs and psychological distress symptoms at T2, with interaction analysis of trait resilience during pregnancy.

Outcome		*b* (95% CI)	*p*‐value	*b*_BC_ (95% BCa CI)	*η* ^2^
Mothers, EPDS	Main effects				
	(Intercept)	10.11 (8.04–12.19)	< 0.001	10.13 (7.80–12.42)	
	Resilience T1	−0.21 (−0.25 to −0.16)	< 0.001	−0.21 (−0.26 to −0.15)	0.051
	SLEs (1 event)^a^	1.20 (0.64–1.76)	< 0.001	1.19 (0.65–1.79)	0.024
	SLEs (≥ 2 events)^a^	1.49 (0.94–2.05)	< 0.001	1.49 (0.94–2.06)	
	Education (mid)^b^	−0.36 (−0.97–0.25)	0.247	−0.36 (−1.00–0.26)	0.005
	Education (high)^b^	−0.80 (−1.39 to −0.22)	0.007	−0.81 (−1.40 to −0.21)	
	Age	0.02 (−0.04–0.07)	0.592	0.02 (−0.04–0.07)	< 0.001
	Moderation				
	Resilience T1 × SLEs (1 events)^a^	0.05 (−0.06–0.16)	0.389	0.05 (−0.10–0.19)	0.001
	Resilience T1 × SLEs (≥ 2 events)^a^	0.06 (−0.05–0.17)	0.268	0.06 (−0.06–0.19)	
Mothers, SCL	Main effects				
	(Intercept)	12.31 (10.02–14.61)	< 0.001	12.33 (10.04–14.52)	
	Resilience T1	−0.24 (−0.29 to −0.18)	< 0.001	−0.24 (−0.30 to −0.18)	0.055
	SLEs (1 event)^a^	1.23 (0.61–1.85)	< 0.001	1.23 (0.63–1.89)	0.017
	SLEs (≥ 2 events)^a^	1.24 (0.63–1.85)	< 0.001	1.25 (0.64–1.88)	
	Education (mid)^b^	−0.70 (−1.37 to −0.02)	0.042	−0.70 (−1.40–0.01)	0.003
	Education (high)^b^	−0.52 (−1.17–0.12)	0.112	−0.53 (−1.20–0.15)	
	Age	−0.06 (−0.12–0.01)	0.075	0.06 (−0.11–0.01)	0.002
	Moderation				
	Resilience T1 × SLEs (1 event)^a^	0.02 (−0.10–0.15)	0.698	0.02 (−0.15–0.17)	< 0.001
	Resilience T1 × SLEs (≥ 2 events)^a^	0.05 (−0.07–0.17)	0.423	0.05 (−0.09–0.17)	
Fathers, EPDS	Main effects				
	(Intercept)	11.27 (8.76–13.78)	< 0.001	11.27 (8.67–13.81)	
	Resilience T1	−0.20 (−0.26 to −0.14)	< 0.001	−0.20 (−0.25 to −0.13)	0.064
	SLEs (1 event)^a^	−0.43 (−1.17–0.30)	0.245	−0.44 (−1.08–0.29)	0.033
	SLEs (≥ 2 events)^a^	1.74 (0.91–2.57)	< 0.001	1.74 (0.82–2.78)	
	Education (mid)^b^	−0.45 (−1.18–0.28)	0.227	−0.45 (−1.23–0.31)	0.004
	Education (high)^b^	−0.54 (−1.28–0.19)	0.146	−0.55 (−1.29–0.17)	
	Age	−0.04 (−0.10–0.02)	0.193	−0.04 (−0.09–0.02)	0.003
	Moderation				
	Resilience T1 × SLEs (1 event)^a^	0.05 (−0.10–0.19)	0.517	0.05 (−0.08–0.22)	0.001
	Resilience T1 × SLEs (≥ 2 events)^a^	0.01 (−0.14–0.16)	0.904	0.01 (−0.16–0.16)	
Fathers, SCL	Main effects				
	(Intercept)	9.34 (6.89–11.79)	< 0.001	9.32 (6.99–12.28)	
	Resilience T1	−0.13 (−0.19 to −0.07)	< 0.001	−0.13 (−0.19 to −0.08)	0.030
	SLEs (1 event)^a^	0.01 (−0.70–0.73)	0.972	0.01 (−0.61–0.76)	0.020
	SLEs (≥ 2 events)^a^	1.45 (0.64–2.25)	< 0.001	1.44 (0.55–2.51)	
	Education (mid)^b^	−0.18 (−0.89–0.54)	0.628	−0.18 (−0.93–0.62)	0.004
	Education (high)^b^	−0.55 (−1.26–0.17)	0.135	−0.55 (−1.27–0.12)	
	Age	−0.08 (−0.14 to −0.02)	0.006	−0.08 (−0.14 to −0.03)	0.012
	Moderation				
	Resilience T1 × SLEs (1 events)^a^	0.04 (−0.10–0.18)	0.553	0.04 (−0.09–0.18)	0.001
	Resilience T1 × SLEs (≥ 2 events)^a^	0.02 (−0.13–0.16)	0.826	0.02 (−0.14–0.16)	

*Note:* The table only displays estimates for the interaction term in the moderation analysis. The moderation analysis included the same variables as the main effects model. Estimates from the linear models (*b* [95% CI]) and from bias‐corrected models (*b*_BC_ [95% BCa CI]) are shown.

^a^ The reference level is 0 event.

^b^ The reference level is low.

### 3.4 Post Hoc Analysis

In the main analysis, SLE was categorised due to the insufficient number of events in the dataset for reliable analyses with other divisions. However, post hoc analyses were performed using categories of 0–1, 2–5, and 6+ events. In mothers, a significant interaction effect was observed for 6+ SLEs compared to the reference category of 0–1 events (*b* = −0.49, 95% CI [−0.82; −0.16], *p* < 0.001). In addition, an interaction effect was observed when comparing the 2–5 category with the 0–1 category, but it did not reach statistical significance (*b* = 0.094, 95% CI [−0.01; 0.19], *p* = 0.079). Caution is warranted in drawing conclusions due to the small sample size of mothers with six or more SLEs (*N* = 18). Unfortunately, a similar analysis could not be conducted for fathers due to the small sample size reporting over 6 SLEs (*N* = 5).

### 3.5 Gender Differences

While the present study did not set out to investigate gender differences, a brief description of the main differences observed is provided to facilitate understanding of the context. The findings indicated that mothers exhibited slightly lower resilience in comparison to fathers (T1: *t*(1231.62) = −4.10, *p* < 0.001; T2: *t*(1353.89) = −2.41, *p* < 0.05). Furthermore, mothers exhibited higher levels of depressive symptoms (T1: *t*(2039) = 7.46, *p* < 0.001; T2: *t*(1405.72) = 4.55, *p* < 0.001) and anxiety symptoms (T1: *t*(1512.89) = 4.57, *p* < 0.001; T2: *t*(2029) = 5.14, *p* < 0.001). In addition, mothers reported more experiences of SLEs (*t*(2043) = 5.96, *p* < 0.001). Mothers were also generally younger (*t*(2043) = −7.35, *p* < 0.001) and more highly educated (*t*(1289.31) = 3.60, *p* < 0.001) than fathers but had lower net incomes (*t*(1153.98) = −9.68, *p* < 0.001). Detailed means and standard deviations for these variables are provided in Table 1. The regression analyses yielded consistent results for mothers and fathers, with one notable exception: in mothers, both one and two or more SLEs were associated with increased symptoms of psychological distress, while in fathers, only two or more SLEs were associated with heightened symptoms of distress.

## 4 Discussion

The objective of the study was to examine the stability of parents' trait resilience and to investigate its associations with SLEs and distress symptoms from pregnancy through early parenthood over a 6‐year follow‐up period. Notwithstanding the period of change, challenge, and potential adversity, the trait resilience of parents exhibited stability to some extent over time and through various life events, as postulated by the assumption of the first hypothesis (H1). In particular, a significant proportion of parents with the lowest or highest trait resilience demonstrated consistent levels of resilience at the 6‐year follow‐up. To the best of our knowledge, this is the first study to investigate trait resilience with a longitudinal model that considers this level of stability from different perspectives. The mean scores indicated a slight improvement in trait resilience among both parents. The correlation of trait resilience between the two time points was moderate for mothers (0.54) and strong for fathers (0.66), which are comparable to some previous studies (Ríos‐Risquez et al. 2018; Q. Wang et al. 2022). Furthermore, the trait resilience during pregnancy predicted trait resilience 6 years later. In summary, the results of these analyses indicate that trait resilience demonstrated stability to some extent over the follow‐up period, while also indicating susceptibility to change (H1). Additionally, it was postulated that SLEs could serve as a moderating factor in the association of the two trait resilience factors, potentially attenuating the strength of the association (H2). In contrast with the second hypothesis, the occurrence of stressful life events did not serve to moderate this association. This result could be interpreted as an indication of the stable nature of trait resilience. The third hypothesis (H3) proposed that trait resilience may serve as a protective factor against the psychological distress associated with stressful life events (SLEs). While a higher prevalence of SLEs was associated with increased depressive and anxiety symptoms, baseline trait resilience was associated with lower distress symptoms. However, the findings indicated that trait resilience did not provide additional protection specifically in the context of SLEs. In other words, trait resilience offered a protective effect against distress symptoms, but this effect remained consistent regardless of the number of SLEs experienced.

This finding is inconsistent with the results of two previous studies that identified an interaction effect between resilience and psychiatric (Hjemdal et al. 2006; Sheerin et al. 2018). However, these studies differed from the present study in several ways. Hjemdal et al. (2006) defined resilience in a more expansive manner, incorporating factors such as social competence and planned future. Furthermore, the assessment of SLEs covered a broader range of experiences. In contrast to our study, Sheerin et al. (2018) did not measure resilience as a self‐reported trait but rather through outcomes. Additionally, their study focused on major depressive disorder and generalised anxiety disorder, whereas our study examined milder distress symptoms. Nevertheless, the impact of trait resilience may be more pronounced in contexts characterised by highly stressful situations, as suggested by the findings of our post hoc analysis. Moreover, the specific study population of parents of young children distinguishes our study from the two aforementioned studies. This raises the question of whether, for instance, parenting‐related stressors or protective factors contribute to our findings. Indeed, there is evidence indicating that trait resilience can serve as a protective factor against parental burnout (Sorkkila and Aunola 2022). It is possible that the pervasive presence of trait resilience acts as a general protective factor throughout early parenthood, potentially obscuring specific interaction effects. The finding that trait resilience was most stable among parents with high or low resilience raises the question of whether there are certain thresholds or specific factors that contribute to the maintenance of trait resilience, potentially creating a self‐reinforcing cycle. While there is likely no singular pathway or methodology to enhance trait resilience, it is imperative to acknowledge, firstly, that change is indeed possible and, secondly, that there is also a tendency towards stability. This also highlights the necessity for additional support for parents who report low levels of trait resilience, as spontaneous improvement may be unlikely in this group.

## 5 Limitations and Future Directions

While this study offers valuable insights, it is important to acknowledge the limitations of the study, as they may have influenced the findings and interpretations. The instrument utilised to assess SLEs was not validated. Moreover, the impact of SLEs on individuals can vary considerably, with different events potentially exerting distinct effects. The self‐reported nature of the data may introduce a degree of bias, potentially impacting the reliability of the results. For instance, participants may over‐ or understate their resilience or underreport distress, and over time, their interpretation of the questions may change. The high attrition rate in our longitudinal cohort study may have introduced a selection bias, resulting in a final sample that was, on average, more highly educated, exhibited fewer symptoms, and among mothers, had higher levels of resilience. However, the attrition and study populations were relatively similar in most respects, with only small differences in means. Nevertheless, the underrepresentation of parents with more pronounced symptoms and the overrepresentation of mothers with higher resilience may limit the generalisability of our findings to a broader population. Furthermore, the study population exhibited mild symptom severity overall, and only a few individuals reported high levels of SLEs, which similarly limit generalisability of the findings to populations with more severe symptoms and higher levels of SLEs. The sample's homogeneity, being predominantly Caucasian, also constrains generalisability to a more diverse population as. For example, the availability of resources for managing stressful life events or distress, as well as for enhancing resilience, may differ across cultural contexts. Finally, the sample's socioeconomic status, representative of the average, might affect generalisability, as socioeconomic status may influence exposure to stressors and access to resilience‐building resources. These factors must be carefully considered when interpreting the average levels of the variables and the overall findings of this study. Notwithstanding these limitations, this study presents a unique and extensive sample that includes both mothers and fathers, offering important and novel insights. To the best of our knowledge, this is the first study to investigate trait resilience over an extended follow‐up period with such a high level of detail. Future research should focus on verifying the stability of resilience over time and using more diverse samples to enhance the robustness and applicability of the findings. Moreover, a more detailed analysis of the specific effects of different types of stressful life events on trait resilience and distress would improve our comprehension of these phenomena and facilitate the development of more precise support and intervention strategies. Additionally, it would be advantageous to examine the relationship between trait resilience and outcome‐defined resilience, as there may be some self‐reporting bias in measures of trait resilience. Moreover, an investigation into the association between trait resilience and other variables linked to resilience, such as social support and coping strategies, would be advantageous.

## 6 Conclusion

The findings indicate that, even during the dynamic period from pregnancy to early parenthood, trait resilience demonstrates a certain degree of stability while also exhibiting susceptibility to change. Trait resilience has been demonstrated to have a protective effect against depressive and anxiety symptoms, underscoring its importance for the promotion of mental health and well‐being during this pivotal life stage. As trait resilience can be viewed as an individual's subjective perception of their capacity to cope with adversity, it may serve as a pivotal factor in mental health and social work with parents. It seems reasonable to posit that there are a number of ways in which trait resilience can be built. For those engaged in professional work with families, it would be of the utmost importance to give close attention to the experiences of parents and the strategies they have identified as being effective in the management of adversity. Furthermore, when necessary, assistance should be provided with the identification of resources that could prove beneficial. For example, some may derive benefit from a focus on relationships and social support, while others may find particular cognitive strategies, behaviours, or activities, such as exercise, to be more efficacious. In addition, the findings draw attention to the parents with low trait resilience. The persistence of low trait resilience over a 6‐year period among a substantial proportion of parents, coupled with its association with higher levels of depressive and anxiety symptoms, renders these parents particularly vulnerable. It is therefore imperative that this demographic receive prompt support to enhance their mental health and well‐being. In light of these findings, it would be advantageous to incorporate an evaluation of trait resilience as a fundamental element of a comprehensive mental health assessment.

## Ethics Statement

The study protocol has been approved by the Ethics Committee of the Hospital District of Southwest Finland.

## Conflicts of Interest

The authors declare no conflicts of interest.

## Data Availability

The data that support the findings of this study are available through collaboration but not freely accessible due to Finnish legislation.
